# Spatio-seasonal variation of water quality influenced by land use and land cover in Lake Muhazi

**DOI:** 10.1038/s41598-021-96633-9

**Published:** 2021-08-30

**Authors:** Edovia Dufatanye Umwali, Alishir Kurban, Alain Isabwe, Richard Mind’je, Hossein Azadi, Zengkun Guo, Madeleine Udahogora, Anathalie Nyirarwasa, Jeanine Umuhoza, Vincent Nzabarinda, Aboubakar Gasirabo, Gulnur Sabirhazi

**Affiliations:** 1 State Key Laboratory of Desert and Oasis Ecology, Xinjiang Institute of Ecology and Geography, Chinese Academy of Sciences, 818 South Beijing Road, Urumqi, 830011 China; 2 Key Laboratory of GIS & RS Application Xinjiang Uyghur Autonomous Region, Urumqi, 830011 China; 3 Key Laboratory of Urban Environment and Health, Institute of Urban Environment, Chinese Academy of Sciences, Xiamen, 361021 Fujian China; 4 University of Chinese Academy of Sciences, Beijing, 100039 China; 5 University of Lay Adventists of Kigali (UNILAK), Faculty of Environmental Sciences, P.O Box 6392, Kigali, Rwanda; 6 Joint Research Center for Natural Resources and Environment in East Africa, P.O. Box 6392, Kigali, Rwanda; 7 Department of Geography, Ghent University, Ghent, Belgium; 8 Faculty of Environmental Sciences, Czech University of Life Sciences Prague, Prague, Czech Republic

**Keywords:** Climate sciences, Environmental sciences, Hydrology

## Abstract

Understanding the influence of land use/land cover (LULC) on water quality is pertinent to sustainable water management. This study aimed at assessing the spatio-seasonal variation of water quality in relation to land use types in Lake Muhazi, Rwanda. The National Sanitation Foundation Water Quality Index (NSF-WQI) was used to evaluate the anthropogenically-induced water quality changes. In addition to Principal Components Analysis (PCA), a Cluster Analysis (CA) was applied on 12-clustered sampling sites and the obtained NSF-WQI. Lastly, the Partial Least Squares Path Modelling (PLS-PM) was used to estimate the nexus between LULC, water quality parameters, and the obtained NSF-WQI. The results revealed a poor water quality status at the Mugorore and Butimba sites in the rainy season, then at Mugorore and Bwimiyange sites in the dry season. Furthermore, PCA displayed a sample dispersion based on seasonality while NSF-WQI’s CA hierarchy grouped the samples corresponding to LULC types. Finally, the PLS-PM returned a strong positive correlation (+ 0.831) between LULCs and water quality parameters in the rainy season but a negative correlation coefficient (− 0.542) in the dry season, with great influences of cropland on the water quality parameters. Overall, this study concludes that the lake is seasonally influenced by anthropogenic activities, suggesting sustainable land-use management decisions, such as the establishment and safeguarding protection belts in the lake vicinity.

## Introduction

Water resources and their quality are crucial to human life^1^. Water quality monitoring is important for assessing the value of water for a healthy ecosystem, hygienic environment, domestic and other uses such as recreation, agriculture, mining, and consumption^2^. Therefore, effective and consistent information on water quality is relevant and pertinent for the improvement of water management^3^.

Worldwide, the deterioration of surface water quality due to the increasing effects of human activities and climate change have caused great concern^4,5^. The water quality is endangered by both point and non-point source pollution. Compared to point-sources that have currently been effectively managed, the non-point source pollutants from various land-use types, mostly agriculture and fluvial waters are the foremost source of water quality change^6,7^.

On the other hand, climate change can produce heavy storms by changing rainfall patterns, which affect the elements found in the runoff^8,9^. In aquatic ecosystems, different chemical, biological, and physical factors determine the quality of water^10^. Thus, a specific issue in water quality monitoring lies in the difficulty related to the analysis and measurement of numerous variables, as well as high variability owing to both human and nature-related influences^11^.

With the increase in population, the assessment of water quality induced by land use/land cover (LULC) is an emerging concern due to the trends in water quality in different areas. Consequently, there have been various studies investigating the relationship between LULC and water quality concluding that a significant relationship exists between water quality parameters and land use types at different scales^12–15^. Some studies reported that LULC at the catchment scale has the most important relationship with the quality of water^16,17^ whereas others explained that LULC at the local scales might provide improved clarifications on the variations in water quality parameters^18,19^. Accordingly, it is necessary to consider the spatial scales from local to catchment scale for an effective examination of LULC impacts on water quality.

In particular, most tropical lakes in Africa face pollution problems due to rapidly growing populations associated with greater demand for agricultural land, rising economies, and industrialization^20,21^. These latter have resulted in increased water consumption and wastewater discharge, which causes heavy pollution^22^. Similarly, in Rwanda, aquatic resources are deteriorating owing to domestic, industrial, and agricultural wastes,irrigation return flows; fertilizers; surface run-off; urban development; deforestation, and mining^23^. Of these resources, Lake Muhazi is experiencing a dramatic change in its water quality while it is a major source of water for the neighboring community^20^. Therefore, it is vital to analyze the presence of different elements to maintain the sustainability of the lake.

Traditionally, water quality has been evaluated via measuring physico-chemical, and biological parameters such as dissolved oxygen (DO), pH, turbidity, total solids (TS), temperature, total phosphate (TP), nitrate, biochemical oxygen demand (BOD), and fecal coliform (FC)^24^. The measurement of these parameters employs various indices through mathematical and statistical methods that several organizations have suggested and adopted^25^. Among the various water quality indices, the National Sanitation Foundation related index (NSF-WQI) has become the most effective approach used to collect and process data on water resources to improve management activities^26^. The strength of using this index, as opposed to the evaluation of individual water quality variables, is mainly the ability to reduce the bulk of information into a single value to convey the data in a simplified and understandable manner^27^.

Research on the impacts of LULC on water quality have been previously conducted from different scales^28,29^. However, these studies scarcely have given full consideration to the seasonal differences of water quality parameters while no single study has considered this difference in the study area (Lake Muhazi). Netherless, Mupenzi et al.^30^ showed that the lake is polluted by water flow from mountainsides, the use of agrochemicals in the sugarcane plantations, and other human activities around the lake. Additionally, other studies tracked heavy metals contamination including Cd, Cr, Cu, Fe, Pb, Mn, Zn, along with pH, and temperature in tributary rivers of the same lake^20,31^ and found that the lake's high metal content levels are related to the catchment's land-use practices. By adding chlorophyll-a and transparency to the above-mentioned parameters, the same authors analyzed the nutrient inflows and levels and found that the levels of the nutrient are higher. From the above studies, it is clear that there is a weakness in the provision of comprehensive information on different important parameters including the DO profiles which is very important in the investigation of water quality to better understand nutrient transformations in the lake.

Moreover, in Rwanda where Lake Muhazi is located, we noticed that no single peer-reviewed study has endeavored to inspect the correlation between LULC and water quality indices to provide a quick and simple methodology in the quantification of water quality. The above concerns and drawbacks are discussed in this study to estimate the water quality of Lake Muhazi. Hence, the current study investigates the spatial and seasonal variation of water quality influenced by LULC in Lake Muhazi based on water quality index approach to bridge the gap identified in the literature for effective future water quality monitoring.

This study will hopefully serve as a reference line for further studies concerning water quality monitoring to overcome substantial impacts caused by the poor quality of water. We hypothesized that compared with the rainy season, the dry season would exhibit improved water quality status due to less runoff as a result of less agricultural activities in the watershed while larger cropland areas could result in water deterioration in the nearby sites. The specific objectives of this study are (i) determining the seasonal impacts on water quality in the lake; (ii) mapping the concentration of water quality parameters in the lake; (iii) analyzing the relationship between LULC, water quality parameters, and water quality index based on seasons; and (iv) determining the impact of LULC on seasonal variation of water quality.

## Methodology

### Study area

**Figure 1:**
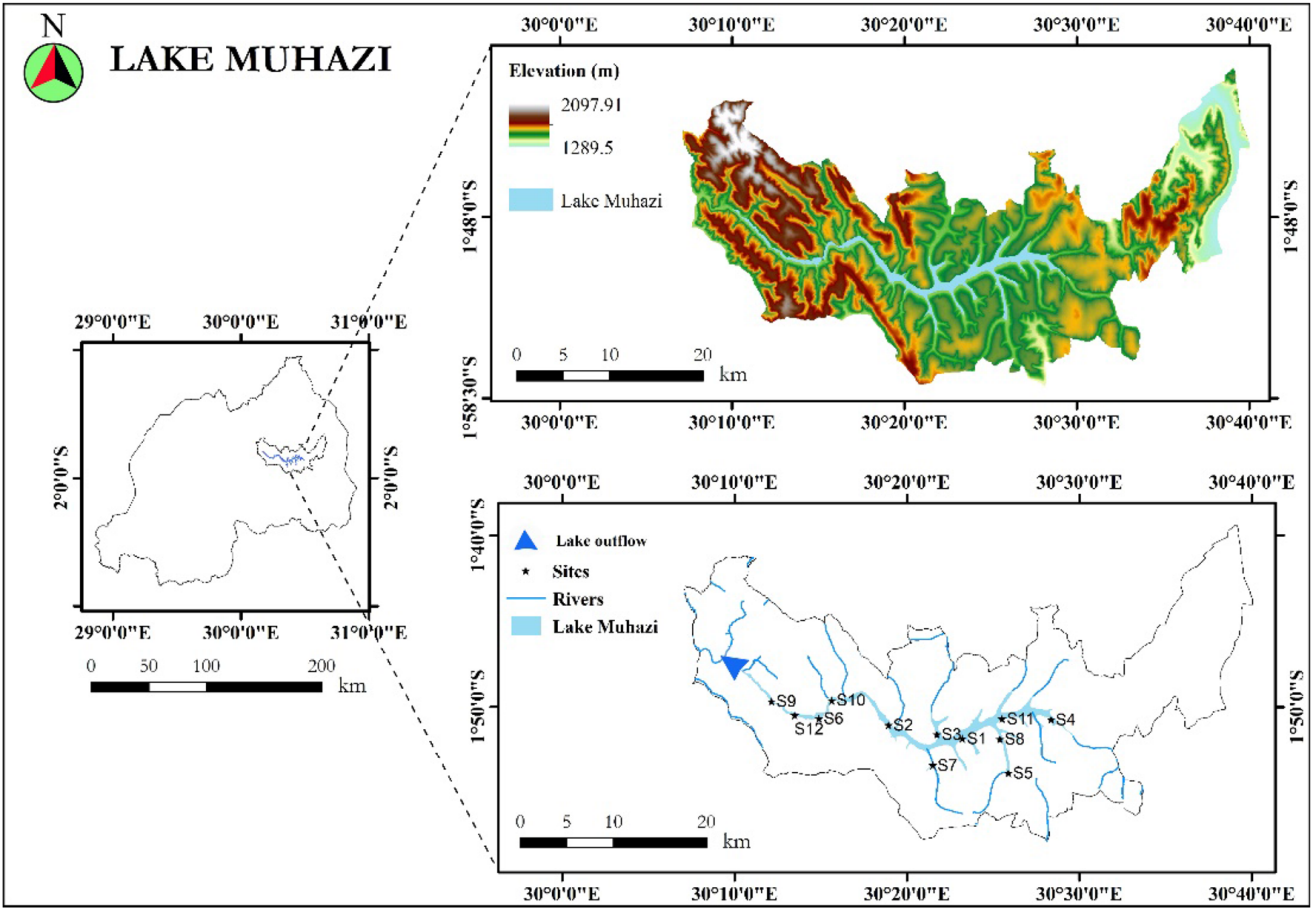
Geographical location of Lake Muhazi and sampling sites along the lake. Source: Authors’ self-implementation with the ArcGIS software version 10.8 (http://www.esri.com). (Map No: GS (2016) 1663).

Lake Muhazi (Fig. 1) is a shallow lake with a long, thin, and snake-like structure and is one of Rwanda's natural aquatic resources. It is situated about 20 km from the eastern side of Kigali, the capital city^31^ and has a shoreline in three of the five provinces of the country. The western side of the lake forms the border between Kigali (Gasabo district) to the south, and Northern Province (Gicumbi district)^32^. In the Eastern Province, two-thirds of the eastern lake forms the boundary between the district of Rwamagana to the south and Gatsibo and Kayonza districts to the north.

It is marked by both urban and rural emissions, especially from rural agricultural activities that are poorly controlled. The lake is located in a mountainous region where the primary activities are farming, ecotourism, and mining. The catchment area is about 830 km^2^ while the lake itself occupies an area of approximately 34.1 km^2^ and a volume of about 3.30 × 10^8^ m^3^^20,31^. The average depth is 10 m (33 ft) with the deepest point of 14 m (46 ft). The climate in the area is warm and humid with annual rainfall ranging between 858 and 1154 mm and the average temperature ranging between 18 and 21 °C.

### Field sampling and sample analysis

Sampling was conducted using the upstream to downstream approach to have a good and clear evaluation of the water quality. Twelve sampling sites (Table 1) were selected for the representation of the entire lake (Fig. 1). The study area has four climatic seasons which are referred as long rainy, short rainy, long dry and short dry. The long rainy season lasts from March to May and the short rainy season from September to November, with an average rainfall of 110–200 mm per month. The long dry season lasts from June and runs to early September and the short dry season lasts from December to the end of February. The average temperature vary between 19 and 27 °C^4^. Therefore, water samples were collected during two field campaigns: one in the dry season (from 20 July to 28 Sep 2018), another in the rainy season (from 12 Oct to 22 Dec 2018). The physico-chemical parameters including dissolved oxygen (DO, mg/L), fecal coliform (FC, CFU/100 mL), biochemical oxygen demand (BOD, mg/L), pH, temperature (°C), total phosphate (TP, mg/L), nitrate (mg/L), turbidity (NTU), and total solids (TS, mg/L) were carried out in situ using a different calibrated instrument with standard solutions and were analyzed using a portable water testing kit (Wagtec, Potalab No 2). These instruments are such as a hand-held conductivity tester EC-1382A (Kelilong Electron) a combined pen-type pH and thermometer combination (ATC Pometer), and YSI Professional Plus hand-held multi-parameter. Water samples were analyzed considering the detection limit and standard technique for water given to us by the laboratory as also specified by the World Health Organization (WHO) and the Rwandan Standards Bureau (RBS). The analysis of major chemical constituents was conducted following the standard laboratory procedures^33^.

**Table 1:** The sampling sites and their geographical coordinate locations.

Site ID	Site names	Coordinates	
		X	Y
S01	Gasave	30.257123	1.513906
S02	Buburankwi	30.185781	1.500955
S03	Umwiga	30.232620	1.509941
S04	Butimba	30.302810	1.495510
S05	Ubwiza	30.291679	1.547498
S06	Gasharu	30.148464	1.494556
S07	Babasha	30.228533	1.539946
S08	Busharu	30.283410	1.514394
S09	Mugorore	30.102641	1.477750
S10	Karambo	30.160822	1.476896
S11	Kibara	30.285485	1.494822
S12	Bwimiyange	30.125038	1.491283

Details on these detection limit can be found at https://www.who.int/water_sanitation_health/dwq/fulltext.pdf and http://www.rsb.gov.rw/fileadmin/user_upload/files/RS_435_revised_2011.docx.

### Land use and land cover (LULC)

To obtain LULC types, a Landsat 8 OLI imagery (data acquisition date: 3 July 2018) with respective scene path and row (172 and 61), at 30 m spatial resolution was acquired from the United States Geological Survey (USGS) EROS data center. Before the classification procedure, the image was pre-processed by performing atmospheric and radiometric correction using the Fast Line-of-Sight Atmospheric Analysis of Hypercube (FLAASH) tool in ENVI software version 5.1 to reduce atmospheric effects and radiometric errors; and hence increase the interpretability and quality of the image. Moreover, during the pre-processing phase, a cloud mask was created to mask out the very few clouds cover in the image^34^. The dataset was then employed to develop an up-to-date LULC map in ArcGIS 10.8 software (http://www.esri.com) using the supervised maximum likelihood classification (MLC) approach. However, satellite imageries cannot be fully corrected as they may contain possible errors leading to uncertainties. For this, the accuracy of the classified LULC map was assessed through the overall accuracy and the Kappa coefficient (Eqs. (1) and (2) respectively) using a total of 216 random points in all land use types sampled from a multi-temporal Google Earth aerial imagery and found the total ground true value data of 201 points. The latter were then overlaid on the classified image for validation. This map was classified into six classes namely, forest, grassland, cropland, built-up, wetland, and water bodies, and the percentage contribution of each land-use types were obtained.

1 $$O.A = \frac{X}{{X}^{^{\prime}}}\times 100,$$

2 $$K= \frac{N{\sum }_{i=1}^{r}{x}_{ii-}{\sum }_{i=1}^{r}\left({x}_{i}+*{x}_{+1}\right)}{{N}^{2 }-{\sum }_{i=1}^{r}\left({x}_{i}+*{x}_{+1}\right)},$$

where O.A represents the overall accuracy, X is the total number of correct samples, X′ is the total number of samples, K is the Kappa index, r is the number of rows in the matrix, x_ii_ is the number of observations in row and column i while x_i_ + and x_+1_ are the marginal totals of row i and column i, respectively, and N is the total number of observations.

### Inverse distance weighted (IDW) interpolation

**Figure 2:**
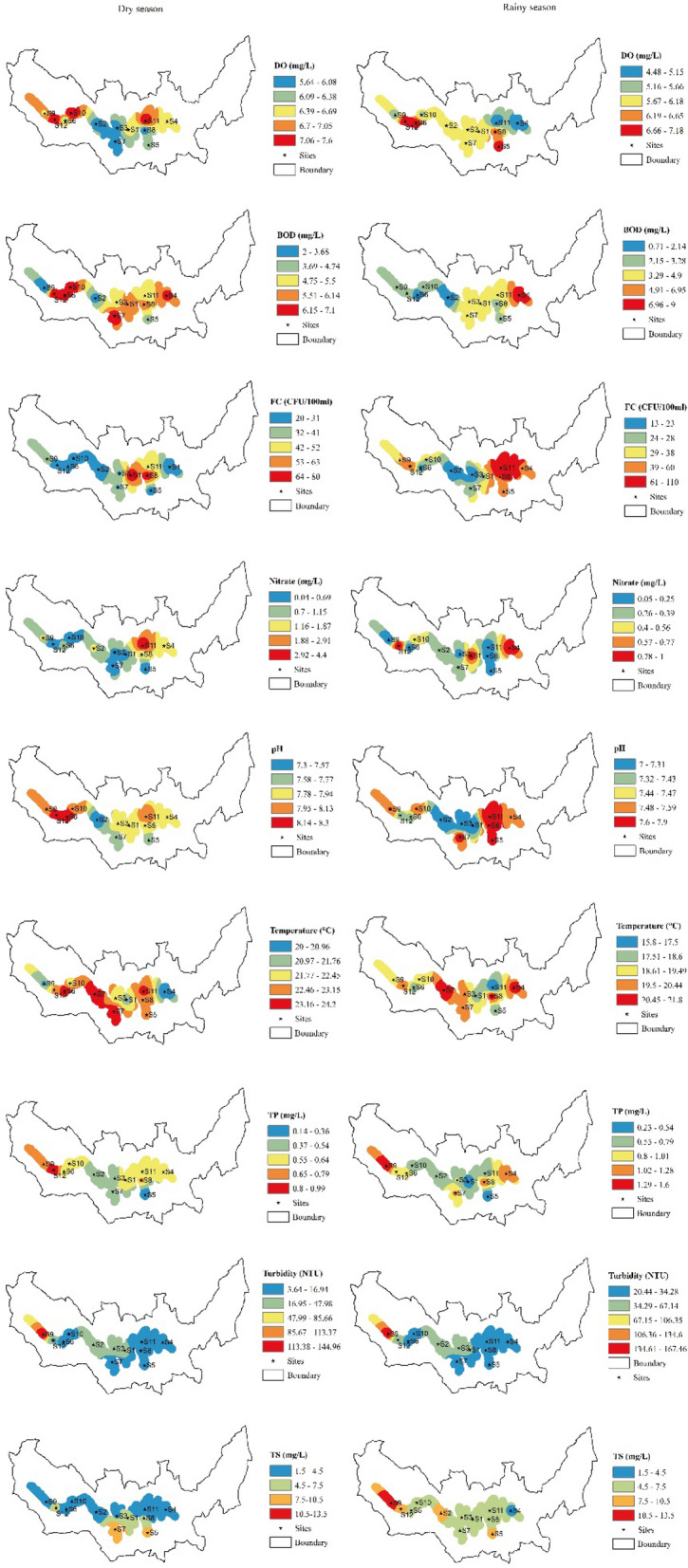
Spatial distribution of the parameters of water quality on Lake Muhazi. Authors’ self-implementation with the ArcGIS software version 10.8 (http://www.esri.com).

As an extension found in numerous spatial analysis tools, IDW allows the interpolation of water quality parameters at an unknown location from known values to create a continuous surface, and understand the scenarios of water quality parameters in the study area^35^. Though there are several spatial modeling techniques including kriging available concerning the application in GIS, the IDW approach has been selected as it enables to process of the spatial distribution of water quality for a comprehensive analysis of the results. IDW was found as the best method to estimate EC and pH, respectively, and an effective tool for determining surface water quality^36^. It is an even reliable and precise method of spatial interpolation to predict the surface water quality in a more accurate format compared to kriging^37,38^. By using ArcGIS 10.8 software, each physiochemical parameter was interpolated with IDW in both seasons to the entire lake (Fig. 2).

However, to control the significance of surrounding points on the interpolated value, the exponent of distance was set to default as “2” which is commonly used considering that the most reasonable results in the IDW technique are obtained using exponents values ranging from 0.5 to 3. The approach was then used to construct spatial distribution maps of the parameters to the entire lake.

### Calculation of water quality index

Among water quality indices, the National Sanitation Foundation Water Quality Index (NSF-WQI) is the most commonly used^39–41^. The significance of numerous parameters using this index varies based on the use of water. Therefore, the used values include the concentration of each parameter in the 12 selected sites in different seasons (dry and rainy). The parameters’ weights (Table 2) have been determined by Brown et al.^42^ in support of the National Sanitation Foundation by requesting the experts to assign values (from 0 to 100) to different concentrations of each of the selected parameters. Hence, Numerical ranges of WQI status were classified into five classes namely Excellent (91–100), Good (71–90), Medium (51–70), Bad (26–50) and Very bad (0–25)^41,42^.

**Table 2:** Weights and sub-index per each parameter.

Parameters	W_i_	S01	S02	S03	S04	S05	S06	S07	S08	S09	S10	S11	S12
**Dry season**													
FC	0.17	47	63	59	63	61	59	58	48	55	58	54	63
pH	0.16	90	93	87	90	92	73	91	87	84	80	80	73
BOD	0.11	50	66	56	49	59	46	47	51	80	48	57	46
DO	0.10	5.0	5.0	5.0	5.0	5.0	5.0	5.0	5.0	5.0	6.0	6.0	6.0
Temp	0.10	20	17	19	22	18	17	16	18	20	19	17	18
TP	0.10	53	66	60	54	94	58	59	52	51	57	58	40
Nitrates	0.10	97	96	96	96	97	96	97	96	96	97	68	97
Turbidity	0.08	61	52	51	75	73	83	89	86	5.0	82	80	70
TS	0.07	80	80	80	80	81	80	81	80	80	80	80	81
**Rainy season**													
FC	0.17	61	65	68	50	52	63	61	50	56	59	43	51
pH	0.16	88	88	88	93	90	93	91	92	92	92	87	93
BOD	0.11	64	97	57	38	66	92	60	68	69	68	64	66
DO	0.10	5.0	5.0	5.0	4.0	6.0	6.0	5.0	5.0	5.0	5.0	4.0	6.0
Temp	0.10	26	19	22	20	26	26	21	20	24	24	30	21
TP	0.10	85	54	47	35	89	46	39	35	30	52	57	45
Nitrates	0.10	96	97	97	96	97	97	97	97	97	97	97	96
Turbidity	0.08	49	39	40	58	58	58	61	55	5.0	56	57	51
TS	0.07	81	81	81	80	81	81	81	81	83	81	81	82

The NSF-WQI is mathematically expressed as:

3 $$NSFWQI={\sum }_{i=1}^{n}{W}_{i}{Q}_{i},$$

where n is the number of water quality parameters considered for the calculation of WQI, W_*i*_ represents the weightage assigned to each parameter associated with Q_i_ which is the sub-index for each water quality parameter. The NSF sub-index values (NSF-Qis) were calculated using the rating curves and calculated by dividing its known concentration in each water sample after analysis and multiplied by 100.

### Statistical analyses

To determine the relationship between all parameters, water quality index, and land-use types, Pearson's correlation analysis was conducted. Multivariate data processing was carried out using PCA and CA techniques to analyze the spatial and seasonal changes. PCA was used to summarize the overall variation in sample grouping by reducing the dimensionality for all water quality parameters (log-transformed, except pH). Nonetheless, as one of the statistical approaches used in water quality analysis, CA is used to categorize entities including sampling sites into discrete different classes based on a given objective. For this, the among-group similarity is minimized while within-group similarity is maximized^43^. In this study, it was used to analyze whether the classification of sampling sites using physico-chemical parameters may be compatible with the NSF-WQI variance, separately in the dry and rainy seasons. Furthermore, PLS-PM is reported to be a soft modeling method applied to model fundamental paths among blocks of parameters^44^. It is preferred by different researchers as it is simple with no strong assumptions regarding the sample size, distributions, and the estimation scale requirement^45^. Its application in this study eased the estimation of the complex cause-effect correlation among LULC variables, water quality parameters in both seasons, and the NSF-WQI values.

Such mathematical studies have been carried out in R (version 4.0.0) (https://www.r-project.org/) supplemented by the packages including *Hmisc*, *ggplot2*, *plspm*, *superheat,* and *factoextra*^46–50^.

## Results

### The spatial and seasonal distribution of water quality parameters in the lake

The spatial distribution and concentration of measured parameters in the lake water samples at different sites for the dry and rainy seasons were assessed (Fig. 2). The highest amount of DO was 7.6 mg/L in the dry season, while the lowest was 4.48 mg/L at S11 (Kibara) in the rainy season. The FC number was the largest (108 CFU/100 mL) at the S11 (Kibara), and the lowest was 13 CFU/100 mL at the S03 (Umwiga), both in the rainy season. The pH value in the lake was the highest (pH = 8.3) at the S06 (Gasharu) in the dry season and the lowest (pH = 7) at S01 (Gasave) in the rainy season. The highest amount of BOD (9 mg/L) was found at S04 (Butimba), and the lowest (0.71 mg/L) was found at S02 (Buburankwi). The temperature of the lake water was the highest (24.2 °C) at the S07 (Babasha) in the dry season and the lowest (15.8 °C) at S11 (Kibara) during the rainy season. The highest amount of TP (1.6 mg/L) was found at S09 (Mugorore) during the dry season, and the lowest was 0.14 mg/L at S05 (Ubwiza). Additionally, the highest amount of nitrate was 4.4 mg/L at the S11 (Kibara), and the lowest amount was 0.04 mg/L recorded at S10 (Karambo), both during the dry season. The results also showed that the amount of turbidity (167.5 NTU) was high at the S09 (Mugorore) in the rainy season and was low (3.64 NTU) at S04 (Butimba) in the dry season. TS was high at S09 (Mugorore) with 13 mg/L in the rainy season and low (1.8 mg/L) at the S06 (Gasharu) in the dry season.

Besides the maximum and minimum, Table 3 displays the quantified results of the water quality parameters where the computed statistical variables such as the mean and standard deviations in both seasons are also shown. However, average FC, TP, Turbidity, and TS were higher in the rainy season compared to the dry season.

**Table 3:** Statistical description of Lake Muhazi water quality parameters during the dry and rainy seasons.

	Dry season			Rainy season		
	Min	Max	Mean ± Std.dev	Min	Max	Mean ± Std.dev
DO	5.64	7.60	6.52 ± 0.71	4.48	7.18	5.99 ± 0.89
FC	20.00	80.00	36.75 ± 20.33	13.00	108.00	41.33 ± 27.00
pH	7.30	8.30	7.90 ± 0.29	7.00	7.90	7.46 ± 0.31
BOD	2.00	7.10	5.42 ± 1.60	0.71	9.00	3.47 ± 2.07
Temp	20.00	24.20	22.50 ± 1.36	15.80	21.8	19.29 ± 1.79
TP	0.14	0.99	0.57 ± 0.19	0.23	1.60	0.83 ± 0.41
Nitrate	0.04	4.40	1.00 ± 1.17	0.05	1.00	0.39 ± 0.39
Turbidity	3.64	145.00	24.52 ± 39.17	20.44	167.50	42.14 ± 40.68
TS	1.80	6.80	4.08 ± 1.53	4.00	13.00	7.30 ± 2.39

### Correlation between LULC, water quality parameters, and water quality index

#### Land use and land cover map

**Figure 3:**
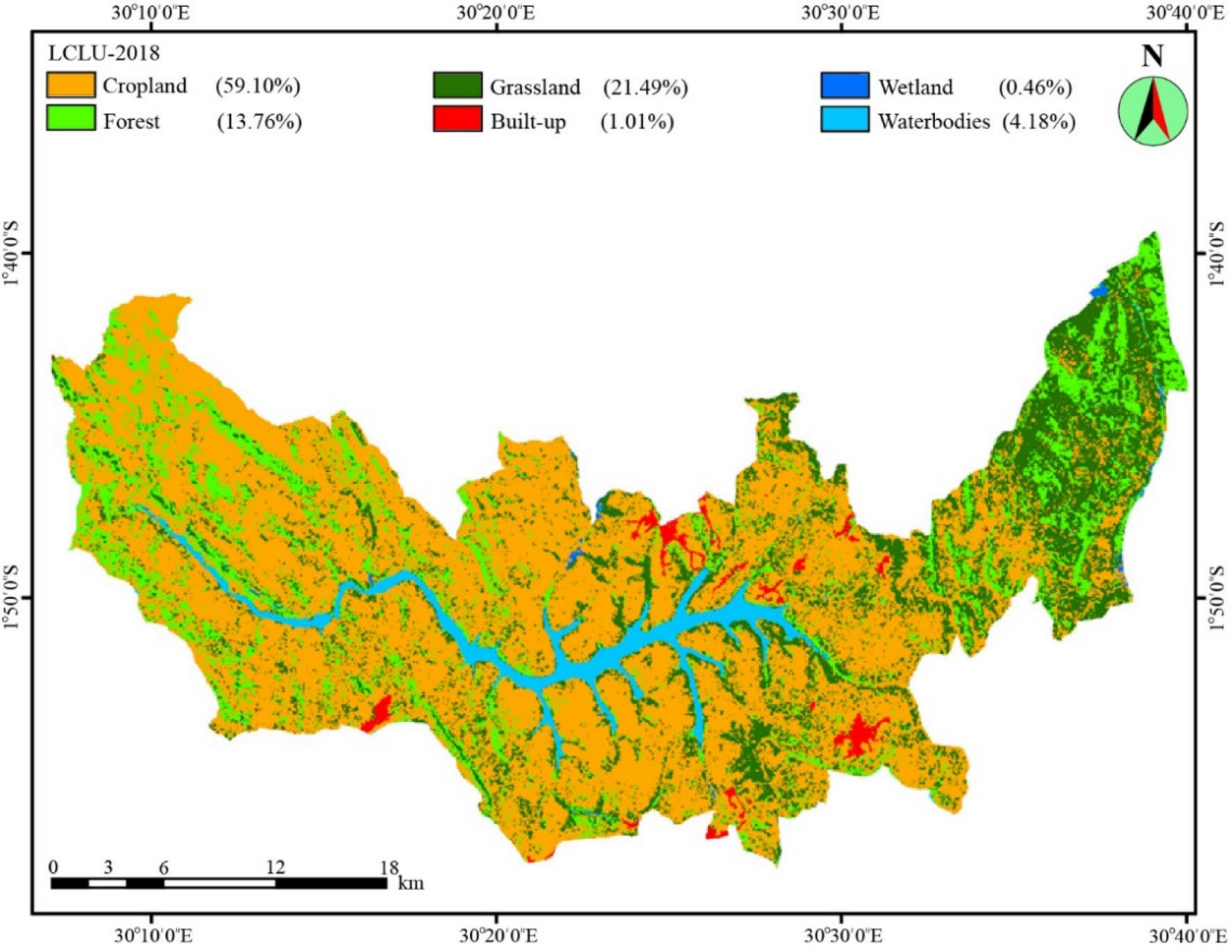
LULC classification map of the Lake Muhazi watershed (2018). Source: authors’ self-implementation with the ArcGIS software version 10.8 (http://www.esri.com).

By percentages, cropland (59.10%), forest (13.76%), grassland (21.49%), wetland (0.46%), built-up (1.01%), and waterbodies (4.18%) were identified around the Lake Muhazi watershed (Fig. 3).

The calculation of the assessed accuracy through the random sampling process for the image revealed an overall accuracy and Kappa coefficient of 93.05% and 91.37%, respectively (Table 4). These values were judged satisfactory for this study. Of all the LULC classes, we noted that cropland covers the highest proportion (59.10%), which explains the availability of different pollutants in the lake coming from agricultural sources. Hence, the classified LULC was worth being used in comparison with water quality parameters per sub-catchments for each site to facilitate the analysis of different correlations.

**Table 4:** Confusion matrices of LULC classification.

LULC types	Cropland	Forest	Grassland	Wetland	Built-up	Water	User accuracy (%)
Cropland	**40**	4	1	0	1	0	86.96
Forest	1	**32**	1	0	0	0	94.12
Grassland	4	2	**56**	0	0	0	90.32
Wetland	0	0	0	**18**	0	0	100
Built-up	0	1	0	0	**37**	0	97.37
Water	0	0	0	0	0	**18**	100
Producer accuracy (%)	88.89	82.05	96.55	100	97.37	100	

### Correlation between LULC types and water quality parameters

Table 5 presents the correlation analysis between water quality parameters and LULC classes based on the seasons. In compliance with^51,52^ the interpretation of Pearson correlation level was followed,where the ranges between 0.5 and 1.0 indicate a strong positive correlation, a medium positive correlation (0.5–0.3, a small positive correlation (0.3–0.1 and a null (0.0–0.09. While values ranging between − 1.0 to − 0.5 (a strong negative correlation, a medium negative correlation (− 0.5 to − 0.3, a small negative correlation (− 0.3 to − 0.1 and a null correlation (0.0 to − 0.09. In the dry season, a strong positive correlation was revealed between DO, TP and cropland; turbidity and forest; FC and built-up and between FC and waterbodies. Moreover A medium correlation between BOD, temperature, nitrate, turbidity, pH and cropland; FC and forest; nitrate, turbidity and grassland and also between FC and wetland while temperature and wetland were negatively correlated. On the other hand, a strong positive correlation was found between BOD and cropland; turbidity and forest; BOD and grassland while TP and built-up showed a strong negative correlation, temperature and built-up. Finally, nitrates and wetland were strongly positively correlated while TP and built-up unveiled a negative correlation. Besides, positive but at a small level and negative correlation between different water quality parameters and land use types were noticed (Table 5).

**Table 5:** Pearson correlation coefficients between LULC types and water quality parameters in the dry and rainy season.

	DO	FC	pH	BOD	Temp	TP	Nitrates	Turbidity	TS
**Dry season**									
Cropland	0.532*	0.257	0.303	0.409	0.445	0.590*	0.349	0.384	0.370
Forest	0.278	0.306	− 0.062	− 0.299	− 0.431	− 0.178	− 0.105	0.579*	− 0.372
Grassland	0.120	− 0.109	0.272	0.158	− 0.436	0.173	0.324	0.460	0.261
Wetland	− 0.082	0.482	− 0.227	0.240	− 0.580*	− 0.097	− 0.266	− 0.178	0.070
Built-up	0.124	0.581*	0.138	0.176	0.013	− 0.140	0.276	− 0.227	− 0.124
Waterbodies	− 0.461	0.637*	− 0.358	− 0.023	0.318	− 0.487	− 0.089	− 0.256	0.416
**Rainy season**									
Cropland	0.215	0.312	− 0.258	0.632*	0.048	− 0.174	− 0.353	0.029	0.262
Forest	− 0.409	− 0.174	0.006	0.198	0.291	0.570	0.169	0.579*	0.270
Grassland	− 0.410	0.467	0.316	0.780**	− 0.050	0.063	0.332	− 0.302	− 0.430
Wetland	− 0.241	− 0.187	− 0.337	0.488	− 0.142	− 0.332	0.524*	− 0.220	− 0.375
Built-up	− 0.029	0.144	− 0.082	− 0.161	− 0.711**	− 0.576*	0.094	− 0.245	− 0.117
Waterbodies	0.385	− 0.081	− 0.044	− 0.398	− 0.25	− 0.507*	− 0.296	− 0.258	− 0.040

*Correlation is significant at the 0.05 level (2-tailed). **Correlation is significant at the 0.01 level (2-tailed).

### The relationship between water quality parameters and water quality index

**Figure 4:**
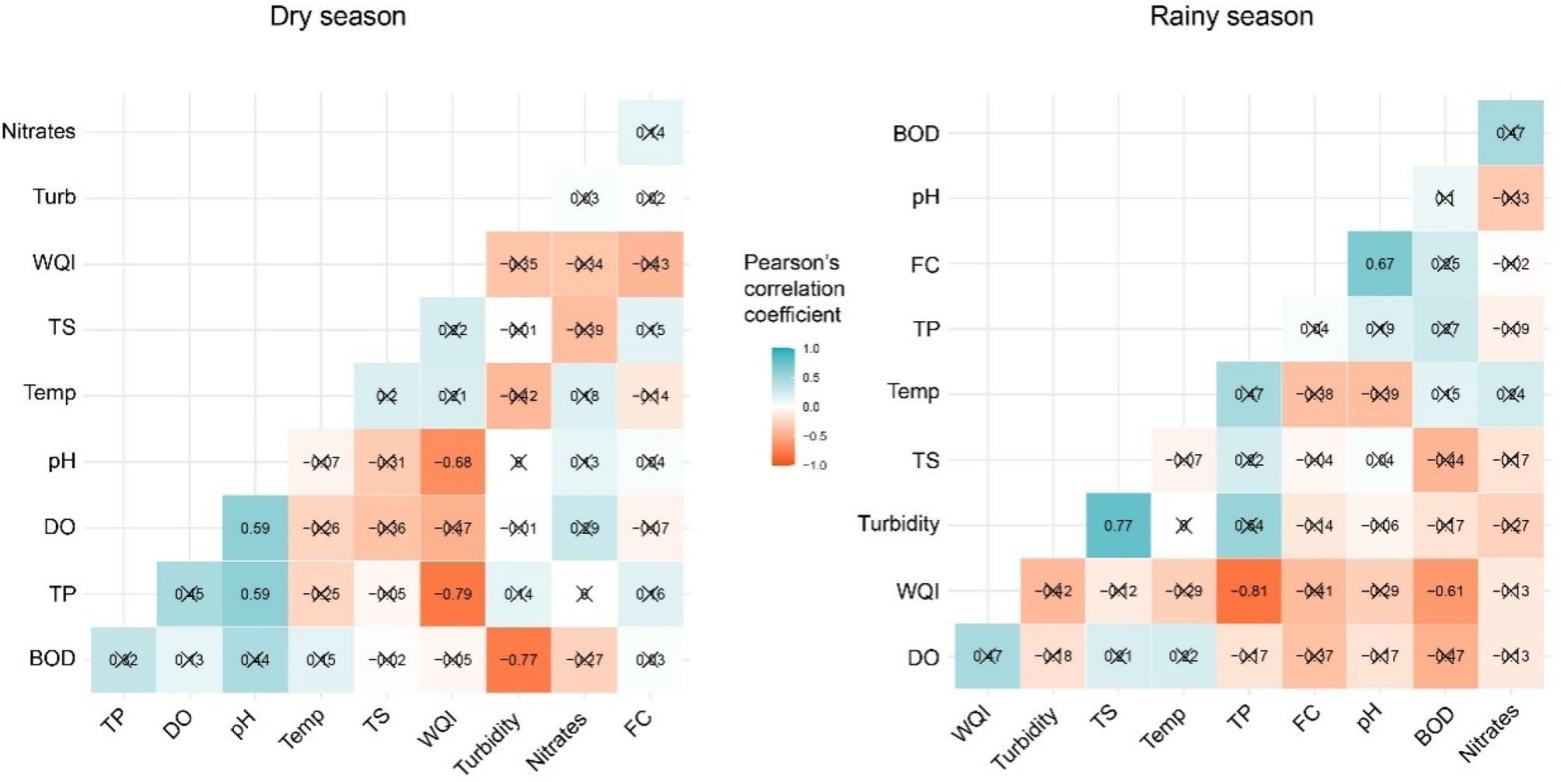
The correlation plot between water quality parameters and the NSFWQI in the seasons. Source: authors’ self-implementation with R software version 4.0.0 (https://www.r-project.org/).

During the rainy season, all sites indicated medium status except S04 and S09 while S09 and S12 indicated bad status during the dry season (Table 6). Nonetheless, the water quality of Lake Muhazi was classified as medium regardless of the seasons. In the rainy season, both the inflow (Butimba) and outflow (Mugorore) showed worse quality than the dry season. The correlation coefficients between parameters for the dry and rainy season and WQI were presented in Fig. 4.

**Table 6:** The water quality status based on the NSFWQI during the dry and rainy seasons in Lake Muhazi.

Site ID	Site name	Dry season		Rainy season	
		WQI	Status	WQI	Status
S01	Gasave	51.25	Medium	57.62	Medium
S02	Buburankwi	56.08	Medium	57.39	Medium
S03	Umwiga	53.20	Medium	53.15	Medium
S04	Butimba	55.02	Medium	48.43	Bad
S05	Ubwiza	59.63	Medium	58.01	Medium
S06	Gasharu	52.72	Medium	58.66	Medium
S07	Babasha	55.30	Medium	53.47	Medium
S08	Busharu	52.79	Medium	51.72	Medium
S09	Mugorore	50.39	Bad	48.83	Bad
S10	Karambo	53.84	Medium	55.34	Medium
S11	Kibara	51.03	Medium	52.80	Medium
S12	Bwimiyange	50.96	Bad	52.69	Medium

### Effects of LULC on the seasonal variation of water quality

**Figure 5:**
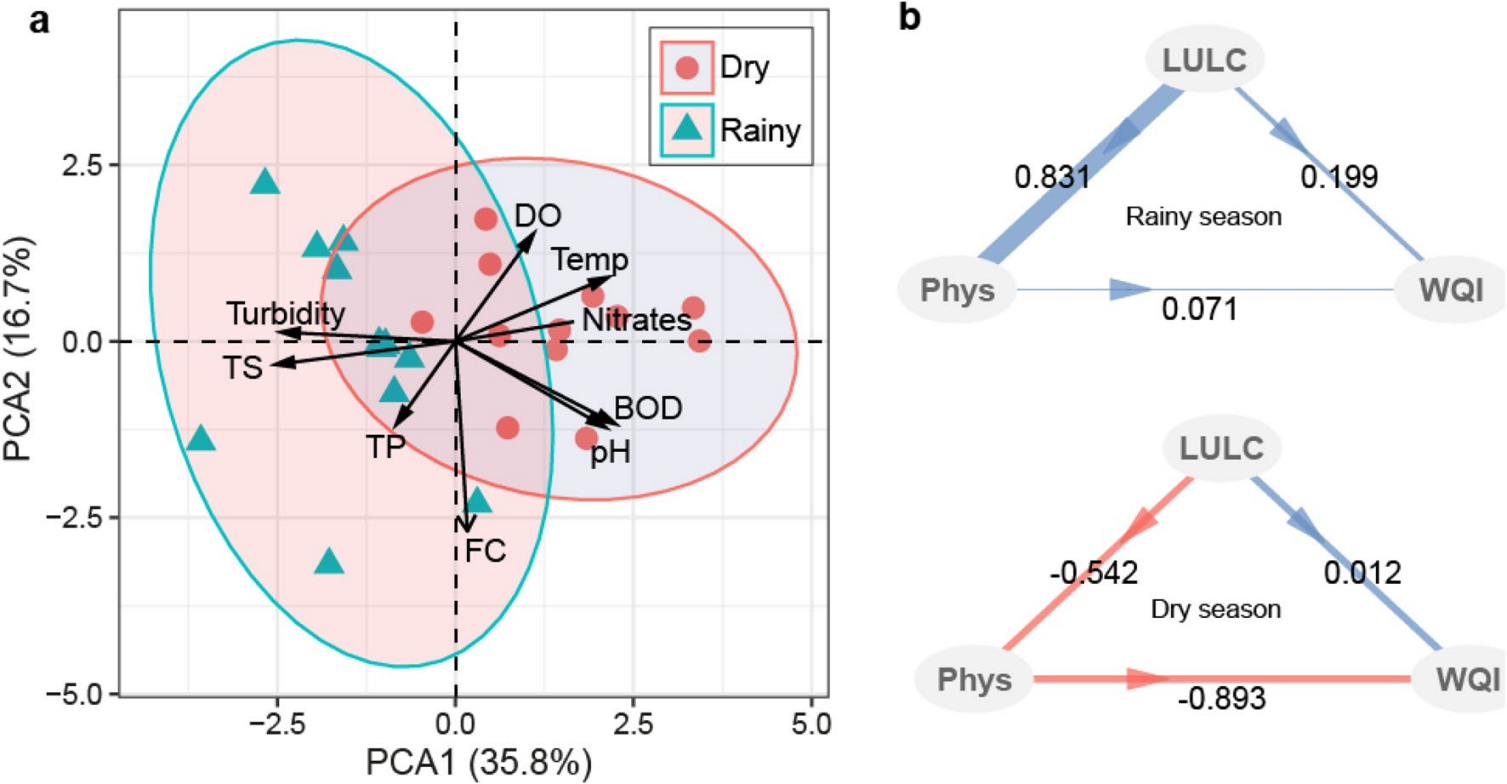
Principal component analysis of all sampled water quality parameters (**a**) and the correlation between land-use types, water quality parameters, and water quality index in seasons (**b**). Source: authors´ self-implementation with R software version 4.0.0 (https://www.r-project.org/).

**Figure 6:**
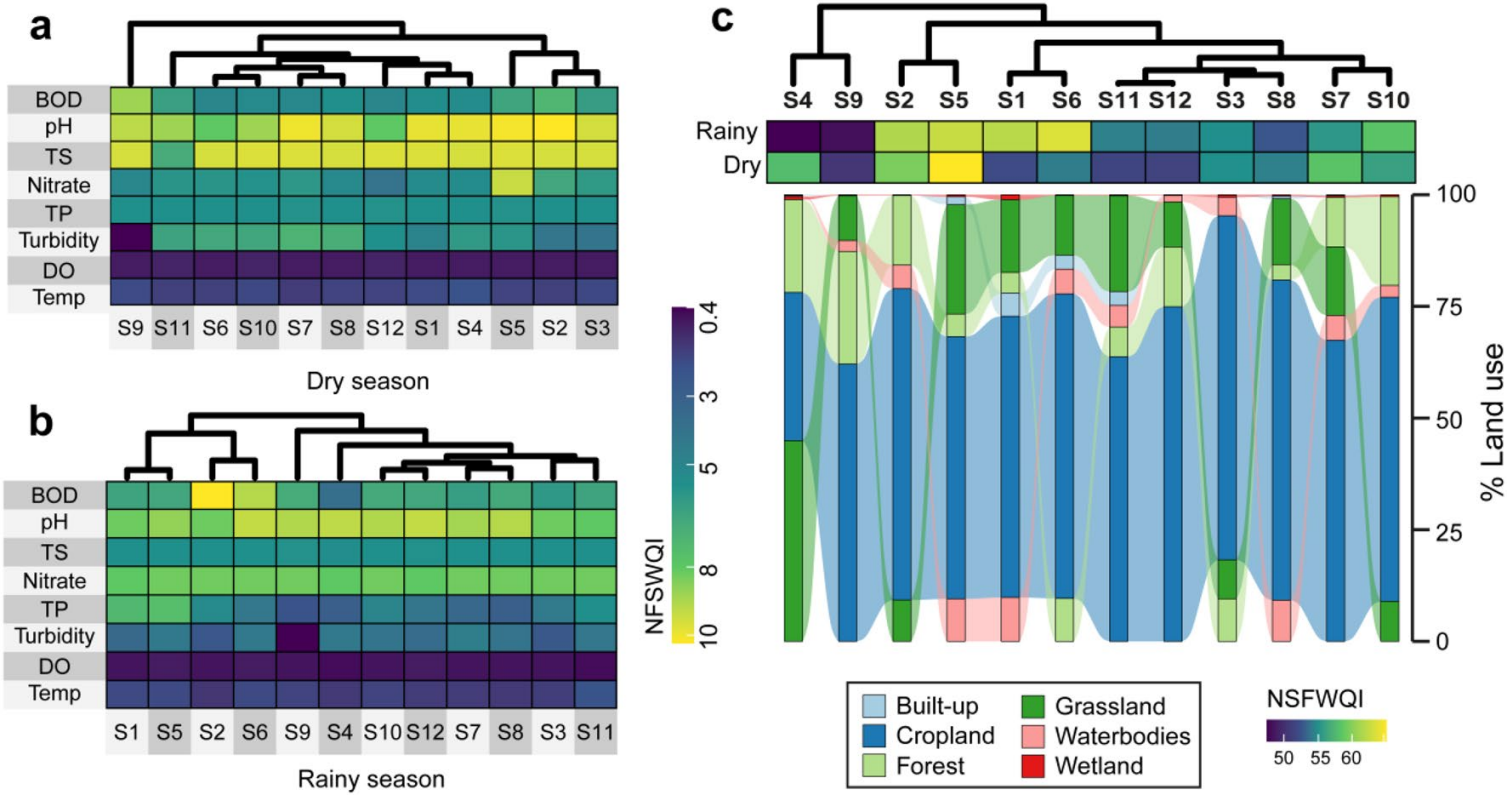
Heatmaps and hierarchical cluster analyses of the water quality parameters in seasons (**a**,**b**) and sample clustering based on NSF-WQI and the percentage of land use types at each sampling site (**c**). Source: authors´ self-implementation with R software version 4.0.0 (https://www.r-project.org/).

The PCA of all water quality parameters showed a sample separation based on seasonality. The first PCA axis correlated with turbidity, nitrate, temperature, and DO. The second PCA axis generally separated both the dry and rainy season samples (Fig. 5a). Similar variations among parameters associated with rainfall such as turbidity and TS increasing toward the rainy season samples were observed. In contrast, water temperature generally increased in dry season samples along with nitrates, BOD, and pH. However, FC varied evenly among the two-season samples. The PLS-PM demonstrated that the associations between the variables of land use types, water quality, and water quality index were all positive in the rainy season. Particularly, a strong positive connection between all land-use types and the water quality parameters was observed (Fig. 5b). This correlation was negative in the dry season (Fig. 5b); water quality parameters had a strong negative association with the water quality index. The hierarchical cluster analysis of water quality parameters (except FC) indicated a strong association between S06 and S10, S07 and S08, S01 and S4, S02 and S03, and the unique clustering of samples from the S09 (Fig. 6a). This isolation of S09 was also observed in the rainy season, a season during which S01and S05, S10 and S12, and S07 and S08 were noted (Fig. 6b). Cluster analysis of NSF-WQI in both seasons returned sample groups corresponding with the dominance of cropland in the lake (Fig. 6c). The goodness of fit was 0.31 and 0.22 for rainy and dry seasons, respectively.

## Discussion

### Water quality evaluation of the lake

The core concept of the WQI is to turn variables into a single numerical value^26^. The results show that the rainfall in the region has a direct influence on water quality and this can be confirmed by the bad quality status of water in the inflow and outflow of the study area. In previous studies, Gradilla-Hernández et al.^39^, and Khodzher et al.^53^ claimed that during the rainy season, high quantities of pollutants enter the lake through the inflow. Water quality drops as a result of the area being located near population centers and due to the entry of microbial contaminants. Human activities generally affect the distribution, quantity, and chemical quality of water resources throughout the sedimentation process^54,55^. Therefore, the quality of water inflows to Lake Muhazi has possibly affected the differences in the quality of water on a certain level. This study classified the water quality status of the lake as medium. The level of water quality deterioration and the extent of the impacts varies based on the area’s characteristics with a wide range of possible changes in LULC. These changes result in the removal of forests, increased cropland (which is positively correlated with different parameters in the lake), substituting grasslands, and urban expansion on a large-scale.

### Seasonal variation of water quality based on parameters

Water quality is crucial to the health security of any ecosystem, which is a determination of the biochemical and physical features of the water^41,56^. For instance, DO was used in this study as an important water quality indicator of seasonal variations. Although oxygen is difficult to dissolve, it is needed by all kinds of life forms in water bodies. The results of this research contradict an earlier study whereby the highest value of DO was observed in the dry season and the lowest value in the rainy season^57^. The possible reason for the result obtained from our study can be attributed to the dilution effect as also found by Umer et al.^58^. The physical characteristic of the study area is related much more where greater photosynthetic events and the reduction in turbidity (Table 3) occur in the dry season and result into the generation of high oxygen amount in the lake.

The greatest biological water parameter is FC (*E. coli*) colonies^59^. They can enter groundwater by a direct release of feces from warm-blooded mammals and birds. Although this type of bacteria does not induce a particular disease directly, its presence in water indicates a low level of sanitation. Using the WHO standards, the quality of drinking water in open wells from zones with low precipitation is in the intermediate risk (100 CFU/100 mL) category during both seasons. The primary sources of FC are animal wastes and runoff in the rainy season. Similarly, a high FC amount was found in the rainy season probably as a result of the livestock effluents process. Confirming the presence of FC bacteria in the lake normally implicates recent fecal contamination, which may create an instant health risk when consumed^60^.

Nevertheless, the best pH range for sustainable aquatic life ranges from 6.5 to 8.5^61^. Sewage into water changes the hydrogen ion concentration in water and becomes more acidic or more alkaline depending on the type of waste and chemical substances contained in it^62^. Thus, the quality of the lake water changes in different sites based on the seasons as the changing concentration of pH is possibly due to the influence of lake water penetration, heavy metal pollution, and high biological activities^63^ while the amount of pH during the dry season is scientifically justified by high water volume and greater water retention. In addition, some researcher has reported that the concentration of pH into the water is influenced by meteorological conditions such as ambient atmospheric warming leading to the variation in water quality^64^. For instance, an increase of pH concentration was noticed in the Rhine and Meuse rivers in the dry season following the warming of water by about 2 °C after the severe drought of 2003^65,66^. In the same study, the lower Mekong River, negative significant correlations were generally found between precipitation and pH. Hence, besides anthropogenically-driven factors, this seems to be verified in our study since the atmospheric thermal condition in the dry season might have influenced the pH concentration.

BOD is the quantity of DO required to cut down organic materials present in a given sample of water at a certain temperature in a given time by aerobic bio-organisms in a water body^67^. According to Lokhande et al.^68^, the decrease in BOD indicates a good water quality while its increase indicates polluted water bodies. In agreement with previous studies, the minimum and maximum amounts of BOD were both recorded in the rainy season^41,69^. It was revealed that an increase in BOD within Lake Muhazi comes from commercial wastewater effluents, agricultural wastes, and household sewage from heavy discharge. As BOD affects the quantity of DO directly in lakes and streams, the higher amount of BOD in the lake justifies the critical reduction in aquatic lives^70^.

Temperature exerts a major influence on biochemical activities^71,72^. However, it also affects the level of DO in water, which directs the types of organisms living in lakes and rivers. As temperatures go far above or below the range, the individuals of the species reduce until none remains present. The link between temperature and DO is a natural process as warmer water becomes more easily saturated with oxygen and it can hold less DO. Therefore, based on the previously described results, the temperature might cause serious effects on the lake in the dry season compared to the rainy season which goes always with a low amount of water temperature^73,74^. For any highly significant seasonal temperature change to be observed, data must be collected from the lake over a longer period.

The excess amounts of phosphate and Nitrate into water cause eutrophication leading to excessive algal blooms^75,76^. As essential elements for plant life, phosphorous and Nitrate concentrations in Lake Muhazi (Fig. 2) are higher in dry season which may be justified by relatively more farming activities executed around the area moving into the lake as runoff. Soil erosion that occurs during floods takes away these parameters from adjacent land and the river banks into the water bodies^77,78^. Cropland influence is explained by inappropriate use of inputs, mostly chemical fertilizers specifically urea, and NPK that is hugely used in the study area. According to Najar and Khan^79^ and Solanki^80^, sometimes, the low amount of nitrate during the dry season happens due to the increase of adaptation by biota owing to their peak growth and the use of plankton or aquatic plants for metabolic activities.

Regarding the NTU parameter, its measurement is frequently applied as water quality indicators depending on the clarity and measured total suspended solids (clay or silt), organic matter, inorganic materials, and decaying material^81^. The increased turbidity in the lake, especially in the rainy season, is generally attributed to high amounts of decaying vegetation; which creates a discoloration of water. Consequently, more unclear water leads to less photic zone and less potential for photosynthetic production. Furthermore, the amounts of NTU found in different sites are caused by the loosened soils from cropland around the area taken away by rain and wind to the lake. The effect of this high turbidity in the lake may cause siltation and sedimentation of the habitat of the fisheries and other aquatic life.

Concerning the TS parameter, the high amount results from forest removal around water bodies^62^. Findings disclosed the highest value of TS in the rainy season and the lowest in the dry season. It is important to note that high TS amount can harm the reproduction level and survival of organisms in water^82,83^. However, very low levels can also cause swelling of cells organism, affecting cell density balance of aquatic life^84^. The mixture of settlement and cropland causes the increase of TS especially in Mugorore site with a district road polluting the lake with runoff.

However, water intended for human consumption and other activities must be free from organisms and concentrations of chemical substances that may be a hazard to health^85^. Unfortunately, Lake Muhazi is still used for different purposes including drinking, domestic uses, or recreation. Thus, based on the concentration of parameters found in the lake compared to the international WHO Drinking Water Requirements. As an aid to enhance the care and perception of water quality, the activities should currently be avoided as the lake needs to be well considered and currently be subjected to the conservation and restoration process by improving techniques for minimizing the concentrations of the parameters.

### The link between of land use types and water quality parameters

One of the most serious threats to water quality is the transition of land use in riparian areas^86^. Anthropogenic activities are directly reflected in land use characteristics^87^. Understanding the correlations between water quality and land-use types assists in identifying the primary threats to the quality of water. These correlations are essential for appropriate management of water quality since they can be used to tackle critical areas of land use and therefore, establish appropriate activities to reduce the load of pollutants^12^. The study found positive correlations between different parameters and land use types (Table 5). Ngoye and Machiwa^88^ associated the lack of vegetation cover, with the presence of different parameters in adjacent waterbodies.

The above relationship was confirmed in this study as the results exhibited a shortage of forest (13.76%) and grassland (21.49%) around the area. Generally, vegetation covers such as forest and grassland around the area affect the concentration of some parameters as both acts as nutrient detention mechanisms and eventually contributor to the betterment of water quality^89^. Yet, a negative correlation was found between forest and FC probably attributed to livestock wastage which is not detained during runoff processes as a result of less vegetation cover. Consequently, it can be said that the less amount of forest and grassland in the area play a role in the concentration of pollutants in the lake.

Researches have shown that the urban environment offers a realistic influence on the deterioration of water quality in all parameters^90,91^. The main factor determining the negative impact of development on water quality is the level of impervious surfaces directly connected to a stream. The above can justify the concentration of different parameters found in the lake (Fig. 2) and implicates that built-up land probably plays an important role in the pollution of the lake. The reason for this relates to the fact that built-up areas and their associated increase in impervious surface facilitate the transport of more pollutants by overland flow particularly in rainy season^92^.

However, cropland was the dominant type of land use with 59.10% of the total area. Consequently, the inappropriate use of input fertilizers is the major cause of water quality deterioration of Lake Muhazi. In crop management and production, nutrient changes in water quality are due to the use of fertilizers (organic or inorganic) at a higher rate than those developed by soil particles. As the primary cause of non-point source pollution, fertilization activities dominant in farming activities is one of the main crop management practices that have been reported to be the core nutrients ‘source especially nitrate and phosphorous amount into surface water. The rainfall following the fertilization period in the study area (when crops are at their peak growing phase in the long and short rainy season) results in amplified overflow toward the lake either in solution or linked with eroded soil particles. Further, the transfer of the aforementioned nutrients from fertilization to the lake takes place during subsurface drainage or base flow. The management of fertilization techniques performed in the area to lessen fertilizer release into the lake leads to the major impacts that they are used in the specific site with an increased ability to deteriorate water quality. In this case, the application of fertilizers or manure without integration can trigger the persistent nutrient dispersal and cause intrusion of the lake water into acquifer^93^. The above discussion justifies the correlations between water quality and cropland type which is indicated by the major influence in the increase of water pollution because of the increased influence of agricultural runoff^94^. In conjunction with earlier studies, the concentration of parameters in a water body as a result of LULC depends on the land use planning of an area. Considering the effects of distance from the lake can improve the water quality change^95,96^. Huang et al.^97^ suggested that the different land use types exclusively croplands and built-up should be put to quite some distance away from the water bodies. For this, a set of equidistant buffer zones of some distances to sampling sites can support the understanding of the impacts of land-use types on the concentration of different pollutants because the association between these types and the water quality parameters may change as the distance to water body increases. Therefore, the creation of riparian buffer zones particularly with vegetated areas located adjacent to the investigated lake is one way to effectively lessen the concentration of the measured nutrient in the lake. Thus, increasing the vegetation within large buffer zones should be considered to reduce the concentrations of different parameters in the lake.

### Uncertainties

Uncertainties in water quality-related researches are unavoidable due to difficulties in the identification of the best approach as well as the quality of input datasets under different required conditions^98^. Regrettably, the collection and analysis of water quality data are expensive and require careful handling and analysis in laboratories. Thus, data on these types of research are often scarce to support the model and this contributes to possible uncertainties^99^. For instance, the lack of streamflow data as in this study could be significant in water quality assessment as the concentration of parameters could have been coupled with the collected data to assess the transport mechanisms of different nutrients.

Moreover, data processing and management can also present a certain level of uncertainty. Results may be limited by missing data, methods adopted to estimate missing values, and errors in data management. This study did not address this source of uncertainty as it did not arise from random statistical variation but rather from individual mistakes or equipment faults. Another important uncertainty of the lake water quality data is associated with the sampling measurement. This uncertainty relates to the selection of representative sampling sites as they can affect the optimization of a water sample. The sampling site choice might have substantial effects on the measured concentration of a given parameter considering that some parameters have high spatio-temporal variability within the cross-section of a given reach. Therefore, high-frequency sampling efforts at multiple sampling stations, are needed to get robust results for the whole lake. Nevertheless, the effect of these above-mentioned possible uncertainties is considered not significant for this study since the significance of this study is not to represent an exact condition of the area’s pollution level but to guide the comprehension of the possible future scenarios for policymakers. Hence, reducing uncertainties to improving the understanding of the vulnerability of water quality in light of land use types should be prioritized in future researches.

## Conclusion

Using GIS analysis showed the spatial distribution and the condition of water quality of the Lake. It has been shown that Lake Muhazi is influenced by anthropogenic activities driven by LULC of the area. Furthermore, the condition of the lake’s water quality varies depending on the seasons. From the overall obtained results, the following conclusions and recommendations were drawn:

1. Based on our analysis, water pollution was caused by the increase in human activities especially agriculture around the area.
2. Of all the sites, none has shown an excellent or good status of water quality, therefore water should be properly treated before usage.
3. To retain the runoff before releasing it into the lake and minimize water pollution, rainwater retention ponds can be constructed near developed zones around the study area.
4. The study area needs effective land use planning and formulates non-point source pollution control policies taking into consideration the increase in forest and grassland.
5. It will be significant to consider buffer-zone scales in assessing the effects of land use on water quality in the areas by controlling the anthropogenic activities within and around the watershed by taking necessary actions against polluters.
6. The study is suggested considering the runoff flow direction or incoming flow from the lake Feeder Rivers as part of the strategic sampling approach in future studies.

We believe that this study contains sufficient information to keep making efforts for strong monitoring of pollutants sources. An epidemiological study factor is required in future studies to evaluate the spatial distribution and association analysis between waterborne diseases and the concentrations of different parameters.
